# Primary Care: The Actual Intelligence Required for Artificial Intelligence to Advance Health Care and Improve Health

**DOI:** 10.2196/27691

**Published:** 2022-03-08

**Authors:** Winston R Liaw, John M Westfall, Tyler S Williamson, Yalda Jabbarpour, Andrew Bazemore

**Affiliations:** 1 Department of Health Systems and Population Health Sciences University of Houston Houston, TX United States; 2 Robert Graham Center for Policy Studies in Primary Care Washington, DC United States; 3 Centre for Health Informatics Cumming School of Medicine University of Calgary Calgary, AB Canada; 4 Center for Professionalism and Value in Health Care American Board of Family Medicine Washington, DC United States

**Keywords:** artificial intelligence, primary care

## Abstract

With conversational agents triaging symptoms, cameras aiding diagnoses, and remote sensors monitoring vital signs, the use of artificial intelligence (AI) outside of hospitals has the potential to improve health, according to a recently released report from the National Academy of Medicine. Despite this promise, the success of AI is not guaranteed, and stakeholders need to be involved with its development to ensure that the resulting tools can be easily used by clinicians, protect patient privacy, and enhance the value of the care delivered. A crucial stakeholder group missing from the conversation is primary care. As the nation’s largest delivery platform, primary care will have a powerful impact on whether AI is adopted and subsequently exacerbates health disparities. To leverage these benefits, primary care needs to serve as a medical home for AI, broaden its teams and training, and build on government initiatives and funding.

## Introduction

As noted in a 2019 report on artificial intelligence [1], Yuval Noah Harari wrote, “Humans were always far better at inventing tools than using them wisely” [2]. As data grow exponentially, this maxim is proving to be prescient in health care. From electronic health records (EHRs) and claims to smart devices, there are more electronic data than nucleotides in our individual DNA. Many note the potential of these data to advance the quintuple aim of better patient outcomes, population health, and health equity at lower costs while preserving clinician well-being. Although AI makes meaning from these gigabytes, it will fail without integration with the human and relational intelligence found in primary care.

## A Landmark Report

The power of AI to advance health was highlighted in a recent National Academy of Medicine paper [3]. Its authors note that more affordable devices, broader internet access, and greater demand for digital health allow us to monitor health at home, augment telehealth, and predict which patients will get sick. This report will shape the AI conversation for years to come, and one of its contributions is a catalog of the uses of AI *outside* the hospital. Some examples are listed below:

- Conversational agents are triaging individuals with suspected COVID-19;
- Self-adaptive learning algorithms are working with continuous glucose monitors and insulin pumps to improve glucose control;
- Remote sensors are monitoring vital signs and transmitting data to cloud-based servers where they are acted upon, much like sensors, plugs, and appliances are powering smart homes.

Combining these data with text messages, social media, and geospatial coordinates permits the assessment of moods, outbreaks, and behavior changes. In the conclusion, the authors emphasize that questions on data standardization, usability, and reimbursement remain unanswered and go on to warn that AI can lead to privacy breaches and magnify biases, if diverse stakeholders are not engaged.

## New Era, Same Mistakes

Despite its important insights, it is hard to ignore the absence of one stakeholder group—primary care—where *most* patients get *most* of their clinical care *most* of the time. Providing 50% of ambulatory visits and connecting with public health, primary care is vital to system transformation and needs to play a central role if AI is to enhance value. Prior efforts to integrate technology into health care neglected to engage primary care and resulted in systemic failures [4,5]. For example, family physicians are now spending more time with EHRs than patients, which has contributed to high rates of burnout [6]. Without engagement from primary care, AI could follow a similar path.

Primary care is important for multiple reasons. It is the largest delivery platform in the United States, accounting for 1 in 3 physicians [4,7]. Its presence is powerful enough to reduce mortality [8]. Its EHRs span organs and include behavioral and public health data, providing a comprehensive portrait of individual and population health. Family physicians, in particular, are distributed throughout the country, providing access to rural America [4]. Despite these benefits, only 5% of health care spending is devoted to primary care [9]. This misalignment has led to shortages and fragmentation. Like a wheel without a hub, care is not coordinated without primary care, and patients receive duplicate services and conflicting advice, contributing to greater waste [10,11].

AI has great potential to augment primary care and address these systemic challenges [12]. First, AI assistants can help with the ever-increasing demands for documentation within EHRs, a major factor driving burnout [13]. Using the same technology Alexa employs to turn on lights, play music, and order groceries, virtual assistants can transform speech into notes, and, in the future, can locate relevant information in the EHR, order labs, and adjust medications. By scanning the relevant primary care literature, AI can make recommendations so that patients receive care that is consistent with the current evidence. These innovations should allow primary care clinicians to spend less time locating and entering data and more time attending to patient relationships and solving their problems.

Second, AI can facilitate access to primary care. Conversational agents can interpret symptoms and assist with triage, helping patients to understand whether they need to be seen in the office now, access emergency services, or monitor their symptoms at home. AI can combine this information with data from home devices such as internet-connected scales, glucometers, and, in the COVID-19 era, pulse oximeters to alert clinicians when patients need to be urgently seen. In this way, AI can serve as an early warning system to ensure that patients are evaluated at the right time and at the right place. Smartphones can analyze facial images, alert primary care clinicians when their patients’ moods are deteriorating, and schedule visits before they get worse.

Third, AI can further enable a core feature of primary care—comprehensiveness. Video images can be used to diagnose diabetic retinopathy, dermatologic conditions, and Parkinson disease [14-16]. Applied appropriately, such applications could widen the scope of conditions retained in primary care and ensure that its clinicians are able to operate to the fullest extent of their training. Finally, AI can make care more person-centered. For instance, AI can use meal, geospatial, and activity tracking to provide the personalized health coaching needed to change behaviors and control chronic diseases. These applications would benefit from coordination with primary care so that coaching is reinforced during visits and informed by the patients’ problems and medications. In addition to coaching, AI can predict the risk of acquiring a variety of diseases and identify the specific actions patients can take to mitigate the risk. Innovators in academia and industry are already using AI in these capacities, but more needs to be done to tailor these applications to primary care [12].

Lack of engagement with primary care in the development of these innovations creates a risk of limited implementation or adoption, and even worse, further fragmentation of health care delivery. Data niches could become more entrenched, with relevant information stored in separate locations. Patients could get conflicting information from sensors (eg, an alert indicating that a door is open but a video feed showing that it is closed) and may lack the knowledge to discern which signal to trust. Primary care is well suited to reconcile these conflicts. By eliciting preferences and values through shared decision-making, primary care clinicians help patients make sense of the data and coordinate with all team members (including patients) to refine treatment plans.

## The Missing Link

To avoid additional failures, we propose three recommendations to ensure that primary care is involved in the future of AI.

### 1. In the Home and the Cloud

Primary care clinicians, informaticists, and researchers need to be involved in conversations regarding how health care data are collected, stored, or analyzed, with the primary care practice serving as the medical home for AI. Primary care can inform how data should be stored in the cloud, how algorithms ought to be used to adjust medications, and how tools are best integrated into primary care. This role is important for not only efficiency and effectiveness, but also equity. Because of its broad geographic distribution and focus on what patients need within the context of their communities, primary care enhances equity, offsetting AI’s tendency to widen disparities [17,18].

### 2. Transdisciplinary Teams

To fulfill this responsibility, health informaticists need to be integrated into primary care practices now. Similar to how AI will fail to adapt without primary care, primary care will fail to embrace AI without health informaticists. Team members with backgrounds in health informatics can connect the AI and primary care communities, by helping practices understand the benefits and limitations of AI, integrating AI into existing workflows, customizing AI applications for local contexts, and providing feedback from practices to AI developers. While these changes are daunting, primary care is skilled at adapting to the needs of its patients. As more and more struggled with mental health, primary care integrated behavioral health into their practices. As the number of medications grew, they successfully integrated pharmacists. Similarly, health informaticists can help practices and patients evolve in response to this digital revolution. In the long term, clinicians will need additional training in relevant disciplines, and researchers in other fields will need training in primary care. Ultimately, a transdisciplinary approach is needed to tailor AI to the complexity and longitudinality of primary care [19].

### 3. Government Facilitation

Achieving this vision will require governmental support for data standardization, funding, and governance. The Office of the National Coordinator for Health Information Technology can provide data standardization leadership. The Agency for Healthcare Research and Quality and the National Institutes of Health (NIH) can provide funding for primary care AI research. NIH funding supported a resource that provides data for thousands of patients who stayed in critical care units and that AI researchers use to develop AI tools [20]. A similar data set is needed for primary care. Given the time required to secure funding from the NIH, federal funders should coordinate with foundations and industry partners to stimulate activity in primary care AI now [21]. Finally, because AI needs to be trained and tested in multiple environments, a system that balances collaboration and data governance is needed. Federated learning, where algorithms from collaborators are tested locally, is one such approach, but more is needed to increase its adoption in primary care [22].

## Time Flies Like an Arrow

AI has already transformed our cars, our homes, and our interactions. It has the power to positively impact care delivery, but also the potential to exacerbate existing health system failings. Assuring that AI is translated into knowledge will require engagement from all stakeholders. As a necessary component of AI, primary care is ready to assist.

## Abbreviations

AI: artificial intelligence
EHR: electronic health record
NIH: National Institutes of Health
